# A KETOGENIC DIET REDUCES AGE-INDUCED CHRONIC NEUROINFLAMMATION IN MICE

**DOI:** 10.1093/geroni/igae098.2438

**Published:** 2024-12-31

**Authors:** Mitsunori Nomura, John Newman

**Affiliations:** Buck Institute for Research on Aging, Novato, California, United States; Buck Institute for Research on Aging, Novato, California, United States

## Abstract

Beta-hydroxybutyrate (BHB) is a ketone body synthesized during fasting or strenuous exercise. Our previous study demonstrated that a cyclic ketogenic diet (KD), which induces BHB levels similar to fasting every other week, reduces midlife mortality and improves memory in aging mice. BHB actively regulates gene expression and inflammatory activation through non-energetic signaling pathways. Neither of these activities has been well-characterized in the brain and they may represent mechanisms by which BHB affects brain function during aging. We investigated whether BHB affects brain cells transcriptionally in vitro. Gene expression analysis in primary human brain cells (microglia, astrocytes, neurons) using RNA-seq shows that BHB causes a mild level of inflammation in all three cell types. However, BHB inhibits the more pronounced LPS-induced inflammatory gene activation in microglia. Furthermore, we confirmed that BHB similarly reduces LPS-induced inflammation in primary mouse microglia and bone marrow-derived macrophages (BMDMs). BHB is recognized as an inhibitor of histone deacetylase (HDAC), an inhibitor of NLRP3 inflammasome, and an agonist of the GPCR Hcar2. Nevertheless, in microglia, BHB’s anti-inflammatory effects are independent of these known mechanisms. Next, we examined the brain gene expression of 12-month-old male mice fed with one-week and one-year cyclic KD. While a one-week KD increases inflammatory signaling, a one-year cyclic KD reduces neuroinflammation induced by aging. In summary, our findings demonstrate that BHB mitigates the microglial response to inflammatory stimuli, like LPS, possibly leading to decreased chronic inflammation in the brain after long-term KD treatment in aging mice.

